# Feasibility of Conventional Abdominal Ultrasound for Monitoring Tumor Size After Stereotactic Body Radiotherapy for Hepatocellular Carcinoma

**DOI:** 10.3390/biomedicines14091893

**Published:** 2026-08-25

**Authors:** Masayuki Ueno, Yohei Yamanouchi, Hideki Hanazawa, Hiroyuki Takabatake, Takahisa Kayahara, Youichi Morimoto, Satoshi Itasaka, Hirokazu Mouri, Motowo Mizuno

**Affiliations:** 1 Department of Gastroenterology and Hepatology, Kurashiki Central Hospital, Okayama 710-8602, Japan; ht13066@kchnet.or.jp (H.T.); hm7882@kchnet.or.jp (H.M.);; 2 Department of Gastroenterology and Hepatology, Graduate School of Medicine, Kyoto University, Kyoto 606-8507, Japan; 3 Clinical Laboratory Technology Department, Kurashiki Central Hospital, Okayama 710-8602, Japan; yy10647@kchnet.or.jp; 4 Department of Radiation Oncology, Kurashiki Central Hospital, Okayama 710-8602, Japan; hh17450@kchnet.or.jp (H.H.);

**Keywords:** hepatocellular carcinoma, stereotactic body radiotherapy, ultrasonography, local tumor control, treatment response

## Abstract

**Background/Objectives**: Stereotactic body radiotherapy (SBRT) is increasingly used for hepatocellular carcinoma (HCC) that is unsuitable for surgery, radiofrequency ablation (RFA), or transplantation. Although current evidence for imaging assessment after SBRT is largely based on contrast-enhanced computed tomography (CT) or magnetic resonance imaging (MRI), repeated contrast-enhanced imaging may be difficult to perform at every routine follow-up visit. Thus, we evaluated whether conventional abdominal ultrasound (US) can monitor tumor size after SBRT for HCC. **Methods**: We retrospectively reviewed 67 consecutive patients who underwent SBRT for HCC at our institution between January 2015 and October 2020. After excluding patients treated for local recurrence after RFA or transarterial chemoembolization, those whose lesions were not visible on pretreatment US, and those without follow-up US within one year, 32 patients with 32 nodules were analyzed. Tumor visibility and size changes on US were assessed before treatment and at <6, 6–12, and 12–18 months after SBRT. **Results**: The treated lesion was identified as a discrete nodule on US in 100% (15/15; 95% CI, 78.2–100%), 75.0% (18/24; 95% CI, 53.3–90.2%), and 50.0% (8/16; 95% CI, 24.7–75.3%) of examinations at <6, 6–12, and 12–18 months, respectively. In all cases in which the lesion was no longer measurable on US, contrast-enhanced CT/MRI showed complete or partial response. Local tumor progression occurred in one patient (3.1%) during a median follow-up of 24.1 months; in this patient, interval enlargement was first detected by US 3.7 months after SBRT and was subsequently confirmed by dynamic CT/MRI. **Conclusions**: These descriptive findings suggest that in selected patients with lesions clearly visible on pretreatment US, conventional abdominal US may provide complementary morphologic information during the first year after SBRT when used alongside periodic dynamic CT/MRI. Prospective validation is required before routine implementation.

## 1. Introduction

Hepatocellular carcinoma (HCC) is the sixth most common cancer and a leading cause of cancer-related death worldwide [1]. Treatment is selected according to tumor burden, liver function, and performance status, as described in the Barcelona Clinic Liver Cancer (BCLC) treatment algorithm and other major guidelines [2,3]. For early-stage HCC, surgical resection, liver transplantation, and radiofrequency ablation (RFA) remain the standard curative options, whereas stereotactic body radiotherapy (SBRT) is increasingly used as an alternative for patients who are not candidates for these therapies [2,3,4]. SBRT is particularly useful when RFA is technically difficult, such as for tumors adjacent to major vessels, the liver surface, or the diaphragm [4]. Recent clinical evidence, including a randomized controlled trial and an international practice guideline, has supported SBRT as a standard treatment option for HCC in selected patients [5,6].

Despite this expanding evidence base, assessing the therapeutic effect of SBRT by imaging remains challenging. In contrast to RFA, where the treated tumor usually undergoes immediate coagulative necrosis, the cytotoxic effect of SBRT becomes apparent gradually over several months, and treated tumors frequently retain arterial-phase hyperenhancement for a prolonged period despite being biologically inactive [7,8]. Focal liver reactions in the irradiated field may also mimic or obscure local tumor progression on dynamic computed tomography (CT) or magnetic resonance imaging (MRI). Despite these pitfalls, treatment response assessment after SBRT relies primarily on serial dynamic CT or MRI, preferably interpreted by readers familiar with post-treatment appearances [6,7,8].

In real-world practice, however, performing contrast-enhanced CT or MRI may be difficult at every routine follow-up visit and is not feasible in patients with renal impairment or contrast allergy. Abdominal ultrasound (US) is a radiation-free, low-cost, and repeatable imaging modality that is already widely used to monitor focal liver lesions, yet evidence regarding its role specifically after SBRT is scarce. The only previous reports addressing this question used contrast-enhanced US (CEUS) in small- to moderate-sized cohorts [9,10], and the role of conventional grayscale (B-mode) US has not been formally investigated.

We therefore retrospectively evaluated the longitudinal visibility of SBRT-treated HCCs on conventional abdominal US and explored the potential utility of this method for monitoring changes in tumor size.

## 2. Materials and Methods

### 2.1. Patients

We retrospectively reviewed the medical records of consecutive patients with HCC who underwent SBRT at Kurashiki Central Hospital between January 2015 and October 2020. HCC was diagnosed histologically or clinically based on characteristic arterial-phase hyperenhancement and washout on dynamic CT or MRI. Of the 67 patients (67 nodules) treated during this period, we excluded those who received SBRT for local recurrence after RFA or transarterial chemoembolization (TACE), those whose lesion could not be clearly visualized on pretreatment grayscale US, and those without follow-up US within one year after treatment. Patients treated for local recurrence after RFA or TACE were excluded because prior treatment-related changes, including ablation scars or retained lipiodol, can alter lesion echogenicity and obscure the boundary of viable tumor, making accurate serial size measurement on B-mode US difficult.

All procedures were performed in accordance with the Declaration of Helsinki. This study was approved by Kurashiki Central Hospital, Medical Ethics Committee (approval number 3791). Because of the retrospective design, written informed consent was waived, and informed consent was obtained through an opt-out protocol.

### 2.2. SBRT Procedure

SBRT was delivered as previously described [11]. Patients were immobilized using an individually shaped vacuum pillow and underwent four-dimensional (4D) computed tomography. The gross tumor volume was delineated with the assistance of contrast-enhanced CT and MRI, and an internal target volume was created from the 4D-CT to compensate for respiratory motion; the planning target volume was defined as the internal target volume plus a 5 mm margin. Treatment was delivered using a stereotactic, multi-arc, dynamic conformal technique or volumetric modulated arc therapy with a TrueBeam or Clinac iX system (Varian Medical Systems, Palo Alto, CA, USA), with respiratory gating or breath-holding. Target alignment was confirmed using implanted fiducial markers (Gold Anchor™, Naslund Medical AB, Huddinge, Sweden, or VISICOIL™, IZI Medical Products, Baltimore, MD, USA). A total dose of 40 Gy was delivered in five fractions over five to nine days on an inpatient or outpatient basis.

### 2.3. Ultrasound Examination

Grayscale (B-mode) abdominal US was performed with a convex probe using a commercially available US system (Aplio series; Canon Medical Systems, Otawara, Japan). US was performed immediately before SBRT and during scheduled follow-up. For analysis, follow-up examinations were grouped into three periods relative to the date of SBRT: less than 6 months, 6 to 12 months, and 12 to 18 months. If multiple US examinations were available within a follow-up period, the examination closest to 3, 9, or 15 months after SBRT was selected for the <6-, 6–12-, and 12–18-month periods, respectively. At baseline, the treated lesion was assessed for echogenicity and morphologic features, including the presence of a halo, bright loop, hump sign, nodule-in-nodule appearance, and mosaic pattern. The maximum tumor diameter was measured along the long axis on the largest cross-sectional plane. Static images and cine clips were recorded, and at follow-up, the previous US images were reviewed to reproduce the imaging plane as closely as possible and reduce measurement variability. At each follow-up examination, the treated lesion was classified as identifiable when its margins could be delineated from the surrounding liver parenchyma sufficiently to measure its maximum diameter as a discrete nodule. Lesions that became isoechoic or otherwise indistinct and whose margins could not be sufficiently delineated to measure the maximum diameter were classified as not identifiable. The initial assessment was made in real time by trained sonographers, and US findings were subsequently reviewed and interpreted by experienced hepatologists (M.U., H.T., T.K., and Y.M.).

### 2.4. Definition of Local Tumor Progression

Local tumor progression was diagnosed when tumor enlargement or reappearance/increase in arterial-phase hyperenhancement within the treated lesion was confirmed on dynamic CT or MRI. Dynamic CT or MRI was generally performed at least every six months after SBRT as part of routine surveillance, and additional cross-sectional imaging was obtained when tumor growth, a new lesion, elevated tumor markers, or other findings raised clinical suspicion of progression. No prespecified millimeter threshold on US was used to define local progression; interval enlargement on US was regarded as a finding that could trigger confirmatory cross-sectional imaging. Treatment response of the treated lesion on contrast-enhanced CT or MRI was assessed according to the modified Response Evaluation Criteria in Solid Tumors (mRECIST) on the basis of the change in the diameter of the viable (arterially enhancing) tumor component, and was categorized as complete response, partial response, stable disease, or progressive disease [12].

### 2.5. Statistical Analysis

Continuous variables are expressed as medians with ranges, and categorical variables as numbers with percentages. The primary outcome was the visibility rate of the treated lesion during each of the three follow-up periods, calculated as the proportion of evaluable US examinations in which the treated lesion remained identifiable and measurable as a discrete nodule. Exact 95% confidence intervals (CIs) for visibility proportions were calculated using the Clopper–Pearson method. Because some patients contributed examinations to more than one period, the three visibility proportions are not statistically independent and are presented descriptively. Given the retrospective design, modest sample size, and unequal patient contributions across follow-up periods, all analyses were descriptive; no formal trend or subgroup hypothesis testing was performed.

## 3. Results

### 3.1. Patient and Lesion Characteristics

Of the 67 patients who underwent SBRT for HCC during the study period, 35 were excluded (local recurrence after RFA or TACE, *n* = 26; poor visibility on pretreatment US, *n* = 4; no follow-up US within 1 year, *n* = 5), leaving 32 patients (32 nodules) who met the eligibility criteria for this analysis.

Baseline characteristics of the 32 patients are summarized in Table 1. The median age was 76 years (range: 53–90), and 23 patients (71.9%) were men. The most common etiology of liver disease was hepatitis C virus infection (*n* = 16, 50.0%). Most patients had Child–Pugh class A liver function (*n* = 24, 75.0%), and 17 patients (53.1%) had a modified albumin–bilirubin (mALBI) grade of 1 or 2a, indicating relatively well-preserved liver function [13]. Eighteen patients (56.3%) had BCLC stage 0 disease. The median tumor size was 16 mm (range, 6–30). The most common tumor locations were S7 (*n* = 9, 28.1%) and S8 (*n* = 7, 21.9%). On pretreatment US, 25 lesions (78.1%) were hypoechoic, and a mosaic pattern was present in 9 lesions (28.1%).

### 3.2. Visibility Rate of the Treated Lesion over Time

**Figure 1:**
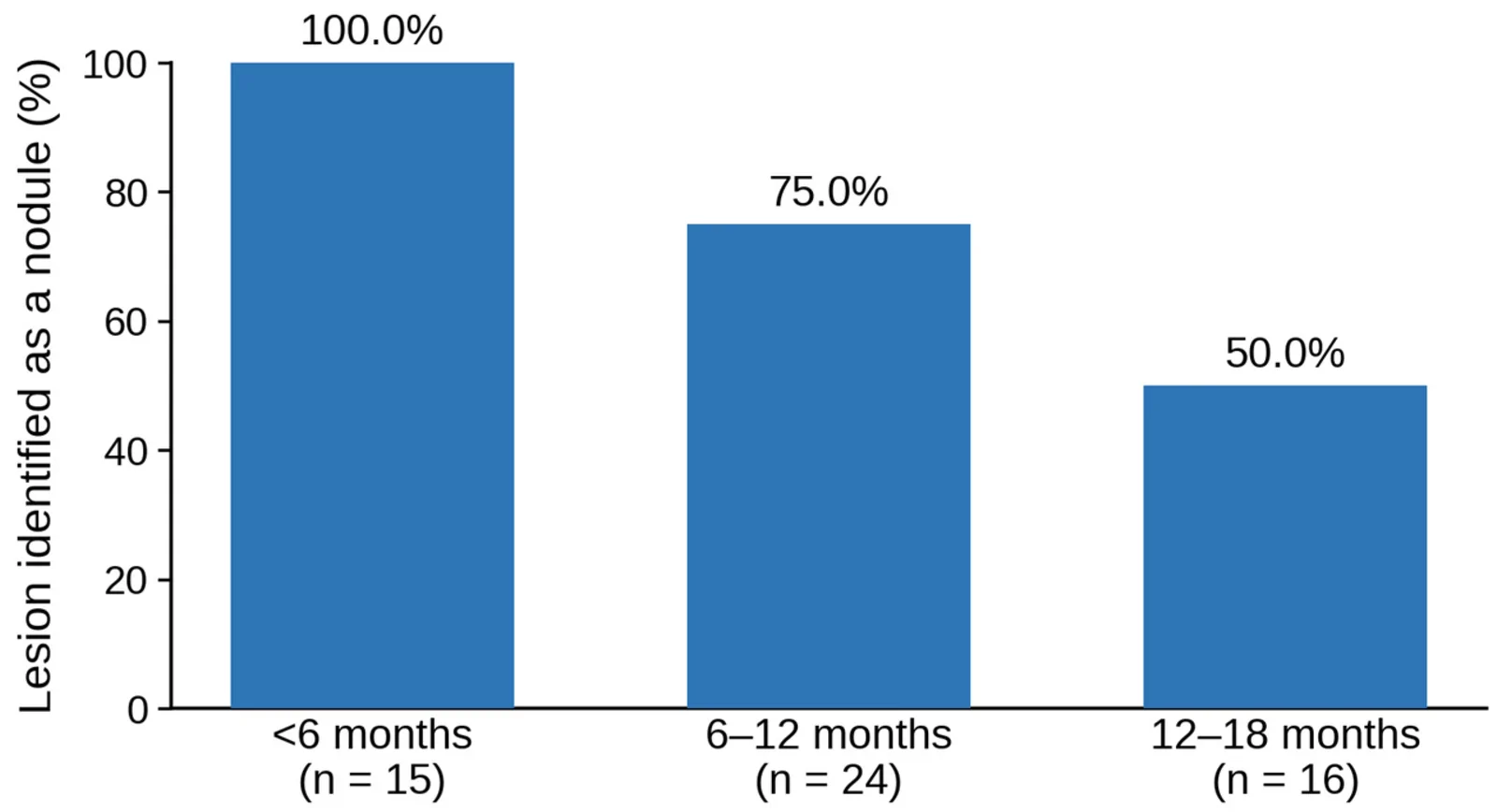
Proportion of follow-up ultrasound examinations in which the treated lesion remained identifiable as a discrete nodule, by follow-up period after stereotactic body radiotherapy.

US follow-up examinations were performed within 6 months, between 6 and 12 months, and between 12 and 18 months after SBRT in 15, 24, and 16 patients, respectively (some patients contributed examinations to more than one period, and not every patient was examined in each period). Among the 32 patients, 13 contributed an examination to one follow-up period, 15 to two periods, and 4 to all three periods. The treated lesion was identified as a discrete nodule in 100% (15/15; 95% CI, 78.2–100%) of examinations within 6 months, 75.0% (18/24; 95% CI, 53.3–90.2%) between 6 and 12 months, and 50.0% (8/16; 95% CI, 24.7–75.3%) between 12 and 18 months (Figure 1 and Table 2). Among the four patients evaluated in all three periods, the treated lesion remained identifiable throughout in three patients; in one patient, it was identifiable during the first two periods but became unidentifiable at 12–18 months. Among the six patients in whom the treated lesion was not identifiable on US between 6 and 12 months, contrast-enhanced CT or MRI showed complete response in four and partial response in two patients. Similarly, among the eight patients in whom the treated lesion was not identifiable on US between 12 and 18 months, contrast-enhanced CT or MRI showed complete response in six and partial response in two patients.

In an exploratory, descriptive subgroup analysis, non-identifiability was more frequent for lesions in the subphrenic segments S7 and S8. Of the 14 follow-up examinations in which the treated lesion was not identifiable, 11 (78.6%) involved lesions located in S7 or S8, and the proportion of examinations in which the lesion remained identifiable was lower for S7/S8 lesions than for lesions in other segments at both 6–12 months (8/13 vs. 10/11) and 12–18 months (3/9 vs. 5/7). By contrast, the distribution of baseline echogenicity among these 14 examinations (12 hypoechoic, 2 hyperechoic) was similar to that of the overall cohort (25 hypoechoic, 7 hyperechoic), suggesting that baseline echogenicity was not a major determinant of the loss of visibility. Because these subgroups were small and follow-up timing was not standardized, these observations are exploratory and were not tested statistically.

### 3.3. Representative Cases of Post-SBRT Tumor Monitoring by Ultrasound

**Figure 2:**
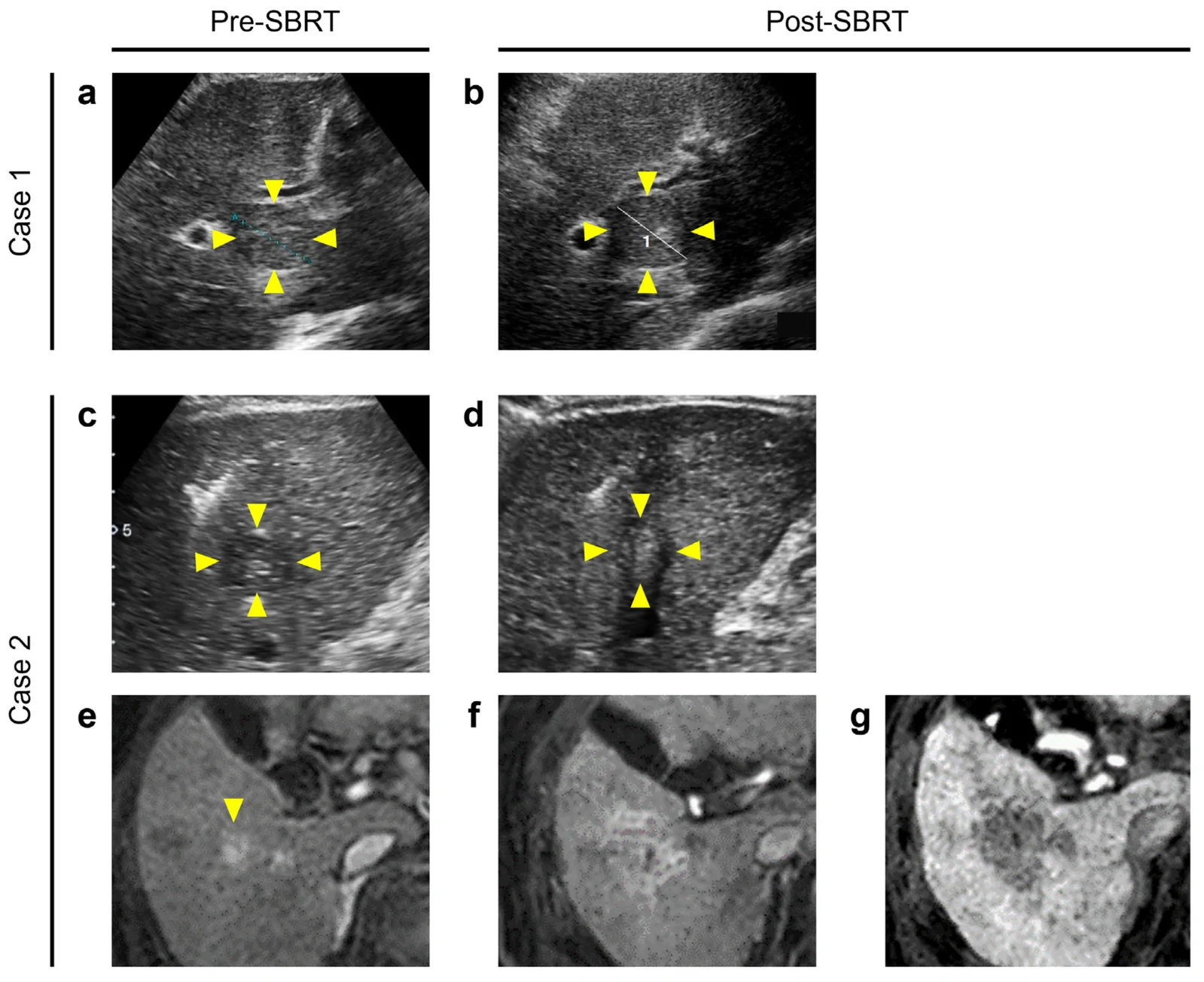
Representative cases of ultrasound (US) monitoring of tumor size after stereotactic body radiotherapy (SBRT). Images obtained before SBRT are shown in the left column (Pre-SBRT), and those obtained after SBRT are shown in the right column(s) (Post-SBRT). On the ultrasound images, the treated lesion is indicated by yellow arrowheads. Case 1 (**a**,**b**): An 87-year-old man with local tumor progression. On grayscale US, the maximum diameter of the treated nodule in segment 8 increased from 30 mm before SBRT (**a**) to 34 mm at 3.7 months after SBRT (**b**), preceding confirmation of progression on dynamic CT/MRI. Case 2 (**c**–**g**): A 60-year-old man with a treated nodule in segment 5. On grayscale US, the nodule remained stable in size after SBRT ((**c**) before SBRT; (**d**) after SBRT), whereas contrast-enhanced MRI showed persistent arterial-phase hyperenhancement (**f**) and hypointensity on the hepatobiliary phase (**g**) of the treated lesion after SBRT ((**e**) before SBRT, with the arrowhead indicating the tumor); these post-treatment findings did not indicate local recurrence but made assessment of tumor size on MRI difficult.

During a median follow-up of 24.1 months (range, 5.7–64.4) after SBRT, local tumor progression was confirmed in 1 of 32 patients (3.1%). This patient, an 87-year-old man with a nodule in S8, underwent follow-up US 3.7 months after SBRT, and the US showed an increase in the maximum diameter of the treated lesion from 30 mm to 34 mm on the largest cross-sectional plane (Figure 2a,b). This interval enlargement was considered suspicious rather than diagnostic of progression and prompted cross-sectional imaging. A similar increasing trend was observed on CT/MRI, which subsequently confirmed local tumor progression, and the patient was treated with salvage RFA.

In another patient, a 60-year-old man with a 15 mm nodule in S5, the treated lesion remained stable in size on US at 9.5 months after SBRT (Figure 2c,d). On contrast-enhanced MRI obtained at baseline, the treated lesion showed arterial-phase hyperenhancement and hypointensity on the hepatobiliary phase (Figure 2e). Post-treatment signal and enhancement changes extended into the surrounding irradiated liver parenchyma, making the lesion boundary and exact tumor diameter difficult to delineate on MRI (Figure 2f,g). In contrast, the lesion margin remained delineable as a discrete nodule on B-mode US, allowing for serial measurement of the maximum diameter and demonstrating no interval enlargement. No local recurrence was confirmed on subsequent follow-up.

## 4. Discussion

In this study, conventional grayscale US identified most SBRT-treated HCC nodules as discrete masses during the first year after treatment. In the single patient with local tumor progression, interval enlargement was detected on scheduled US and subsequently confirmed on dynamic CT/MRI. These descriptive findings suggest a potential complementary role for conventional US in serial morphologic assessment during the first year after SBRT in selected patients whose lesions are clearly visible on pretreatment US.

Previous reports on US after SBRT for HCC have been limited to CEUS. Shiozawa et al. evaluated four patients treated with CyberKnife and described post-treatment hemodynamic changes on CEUS [9]. Funaoka et al. reported that a reduction in tumor vascularity on CEUS was observed in all of the 56 patients without local recurrence by 13 months, with high sensitivity and specificity for distinguishing recurrence from non-recurrence [10]. Both studies relied on dynamic vascular information provided by a contrast agent. Our results suggest that when the treated lesion remains visible, grayscale US can provide serial measurements of its maximum diameter without contrast administration.

The visibility rate of the treated lesion as a discrete nodule fell from 100% within 6 months to 50.0% between 12 and 18 months. This decline may reflect a combination of treatment-induced tumor necrosis and shrinkage and radiation-induced changes in the surrounding liver parenchyma, which can make a lesion progressively less conspicuous or isoechoic [7,8,14,15,16,17,18,19]. Notably, in every examination in which the treated lesion could no longer be identified as a discrete nodule on US beyond 6 months, contrast-enhanced CT/MRI demonstrated at least a partial response (Table 2). Thus, loss of US visibility occurred in the setting of radiologic response on cross-sectional imaging. However, B-mode US alone cannot distinguish whether reduced conspicuity results from tumor shrinkage, post-radiation parenchymal changes, or both.

The patient in whom tumor enlargement was first detected on US illustrates one potential role of B-mode monitoring: interval growth of a treated nodule may serve as a simple trigger for confirmatory dynamic CT/MRI. No specific millimeter threshold for progression was defined on US in this retrospective study, and local progression was diagnosed only after confirmation on dynamic CT/MRI. Because local tumor progression occurred in only one patient, this observation should be regarded as hypothesis-generating rather than as evidence of the diagnostic accuracy of US or of a validated size-increase threshold.

A complementary role was illustrated by the second case. After SBRT, arterial-phase hyperenhancement and radiation-induced changes on CT or MRI can persist for months, extend into the surrounding irradiated parenchyma, complicate lesion delineation, and produce equivocal or false-positive impressions of progression [7,8,14,15,16,17,18,19]. When the treated lesion remains clearly visible on US, serial size measurement may therefore provide additional morphologic information, as in our patient whose lesion margins remained delineable and whose size remained stable on US despite complex post-treatment MRI appearances. Thus, B-mode US may contribute in two complementary ways: by identifying interval enlargement that warrants additional cross-sectional imaging and by providing serial morphologic information when CT/MRI findings are difficult to interpret. Nevertheless, US should be used alongside, rather than in place of, periodic dynamic CT/MRI. The need for caution when interpreting persistent post-SBRT enhancement has also been emphasized in our previous editorial commentary [20].

**Figure 3:**
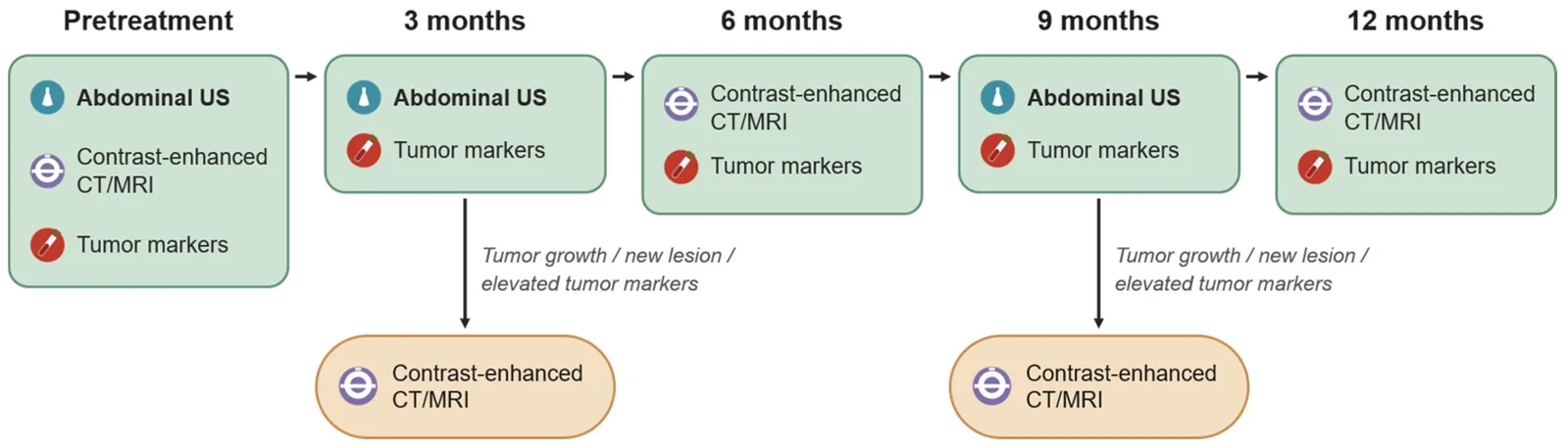
Investigational surveillance framework incorporating conventional abdominal US after SBRT for HCC. Abdominal US is performed before treatment and at approximately 3 and 9 months after SBRT, and contrast-enhanced CT/MRI is performed before treatment and at approximately 6 and 12 months. Additional contrast-enhanced CT/MRI is obtained if tumor growth, a new lesion, or elevated tumor markers are detected. Tumor markers are assessed at each scheduled follow-up visit. This is being evaluated prospectively under UMIN000049003.

Based on these observations, we designed an investigational surveillance framework after SBRT for HCC (Figure 3). A recent EORTC/ESGAR consensus recommends the first follow-up imaging at 3 months after SBRT and repeat imaging every 3 months thereafter, preferably using MRI [21]. Although that consensus favors MRI for follow-up, our framework was designed to preserve this 3-month surveillance time frame while substituting grayscale US for some scheduled cross-sectional examinations and retaining contrast-enhanced CT/MRI at approximately 6 and 12 months. At baseline, abdominal US, contrast-enhanced CT/MRI, and tumor markers are obtained before SBRT. After treatment, tumor markers are assessed every three months, grayscale US is performed at approximately 3 and 9 months, and contrast-enhanced CT/MRI is routinely performed at approximately 6 and 12 months. Additional contrast-enhanced CT/MRI is obtained when US suggests tumor growth, a new lesion is suspected, or tumor markers increase. This framework therefore does not replace periodic dynamic CT/MRI. Because lesion visibility declined substantially after the first year, scheduled US is not proposed beyond 12 months, and the role of routine B-mode US beyond the first year remains uncertain. This investigational framework is currently being evaluated prospectively at our institution (UMIN Clinical Trials Registry, UMIN000049003).

This study has several limitations. First, it was a retrospective, single-center study with a modest number of nodules (*n* = 32). Eligibility required clear lesion visibility on pretreatment US, and patients without US follow-up within one year were also excluded; therefore, the study cohort was enriched for patients in whom US was inherently feasible, and the observed visibility rates may not be generalizable to an unselected SBRT population. In addition, patients treated for local recurrence after prior RFA or TACE were excluded because treatment-related changes could interfere with US delineation, and our findings should not be extrapolated to this clinically important subgroup. Second, the composition of patients differed across the three follow-up periods, with only four patients evaluated in all three periods; therefore, the observed decline in visibility should be interpreted descriptively rather than as a uniform within-patient longitudinal trend. Third, US is operator-dependent. Although examinations were performed by trained sonographers and reviewed by experienced hepatologists, formal interobserver agreement and measurement reproducibility were not assessed, and reproducibility among less experienced readers or at other institutions is unknown. Fourth, because local tumor progression occurred in only one patient, we could not evaluate the diagnostic accuracy of US for detecting local progression or establish a clinically significant threshold for interval size increase. Fifth, although all lesions that became unidentifiable on US showed complete or partial response on contrast-enhanced CT/MRI, B-mode US alone cannot distinguish whether loss of conspicuity reflects tumor shrinkage, radiation-induced parenchymal changes, or both. Finally, baseline echogenicity, tumor location or depth, and other lesion characteristics may affect longitudinal visibility (in exploratory analyses, non-identifiability appeared more frequent for subphrenic S7/S8 lesions than for lesions elsewhere, whereas baseline echogenicity showed no clear association); however, the small sample size, uneven distribution of these characteristics, and non-standardized follow-up in this retrospective cohort precluded reliable subgroup analyses. Larger prospective studies with standardized imaging timing and assessment are needed, and our ongoing prospective study (UMIN000049003) is intended to evaluate these issues more rigorously.

## 5. Conclusions

In this descriptive retrospective cohort, conventional non-contrast abdominal US allowed for serial measurement of many SBRT-treated HCC nodules during the first year among patients whose lesions were clearly visible on pretreatment US. In one patient, interval enlargement on US prompted cross-sectional imaging that confirmed local progression. These findings should be regarded as preliminary and hypothesis-generating, and periodic contrast-enhanced CT/MRI remains essential for treatment response evaluation after SBRT. Prospective larger-scale studies are required to determine whether and how grayscale US can be incorporated as a complementary modality into post-SBRT surveillance.

## Figures and Tables

**Table 1:** Baseline patient and lesion characteristics (*n* = 32).

Characteristic	Value
Age, years, median (range)	76 (53–90)
Male sex, *n* (%)	23 (71.9)
Etiology of liver disease, *n* (%)	
Hepatitis C virus	16 (50.0)
Alcohol-related	5 (15.6)
Hepatitis B virus	4 (12.5)
MASLD	2 (6.3)
Others or undetermined	5 (15.6)
Performance status, *n* (%)	
0	21 (65.6)
1	9 (28.1)
2	2 (6.3)
Child-Pugh score, *n* (%)	
5	12 (37.5)
6	12 (37.5)
7	6 (18.8)
≥8	2 (6.3)
BCLC stage, *n* (%)	
0	18 (56.3)
A	14 (43.8)
Tumor size, mm, median (range)	16 (6–30)
Tumor location (Couinaud segment), *n* (%)	
S1	3 (9.4)
S2	1 (3.1)
S3	2 (6.3)
S4	6 (18.8)
S5	3 (9.4)
S6	1 (3.1)
S7	9 (28.1)
S8	7 (21.9)
Pretreatment US echogenicity, *n* (%)	
Hypoechoic	25 (78.1)
Hyperechoic	7 (21.9)
Mosaic pattern, *n* (%)	9 (28.1)
Halo, *n* (%)	3 (9.4)
Nodule-in-nodule pattern, *n* (%)	2 (6.3)

MASLD, metabolic dysfunction-associated steatotic liver disease; BCLC, Barcelona Clinic Liver Cancer; US, ultrasound.

**Table 2:** Visibility of the treated lesion on follow-up ultrasound and the corresponding treatment response on contrast-enhanced CT/MRI in lesions no longer identifiable on ultrasound by period after SBRT.

Follow-Up Period	Number of Patients	Identifiable as a Nodule on US	Not Identifiable on US	
			CR on CT/MRI	PR on CT/MRI
<6 months	15	15 (100.0%)	0 (0.0%)	0 (0.0%)
6–12 months	24	18 (75.0%)	4/6 (66.7%)	2/6 (33.3%)
12–18 months	16	8 (50.0%)	6/8 (75.0%)	2/8 (25.0%)

SBRT, stereotactic body radiotherapy; US, ultrasound; CT, computed tomography; MRI, magnetic resonance imaging; CR, complete response; PR, partial response. In lesions that could no longer be identified as a discrete nodule on US, the treatment response shown was determined on contrast-enhanced CT/MRI. All such lesions corresponded to complete or partial response on contrast-enhanced CT/MRI. Thus, loss of visibility on US occurred in the setting of radiologic response on cross-sectional imaging.

## Data Availability

The data presented in this study are available on request from the corresponding author. The data are not publicly available because of institutional restrictions related to patient privacy.

## Abbreviations

The following abbreviations are used in this manuscript:

HCC	hepatocellular carcinoma
SBRT	stereotactic body radiotherapy
RFA	radiofrequency ablation
TACE	transarterial chemoembolization
US	ultrasound/ultrasonography
CT	computed tomography
MRI	magnetic resonance imaging
CEUS	contrast-enhanced ultrasound
BCLC	Barcelona Clinic Liver Cancer
ALBI	albumin–bilirubin
mALBI	modified albumin–bilirubin
